# Metabolomics Profiles Associated with the Treatment of Zuojin Pill on Patients with Chronic Nonatrophic Gastritis

**DOI:** 10.3389/fphar.2022.898680

**Published:** 2022-07-11

**Authors:** Xiao Ma, Shuying Xie, Ruilin Wang, Zhongxia Wang, Manyi Jing, Haotian Li, Shizhang Wei, Honghong Liu, Jianyu Li, Qingyong He, Yanling Zhao

**Affiliations:** ^1^ Department of Pharmacy, Chinese PLA General Hospital, Beijing, China; ^2^ School of Pharmacy, Chengdu University of Traditional Chinese Medicine, Chengdu, China; ^3^ Division of Integrative Medicine, The Fifth Medical Center, General Hospital of PLA, Beijing, China; ^4^ Department of Anatomy, Histology and Embryology, School of Basic Medical Sciences, Health Science Center, Peking University, Beijing, China; ^5^ Guang’anmen Hospital, China Academy of Chinese Medical Sciences, Beijing, China

**Keywords:** Zuojin pill, chronic nonatrophic gastritis, efficacy, amino acid metabolism, inflammation

## Abstract

**Objective:** Chronic nonatrophic gastritis (CNG) is the most common digestive disease. In China, Zuojin pill (ZJP) is considered an effective medicine formula for CNG. However, its efficacy and mechanism have never been explored. In order to understand how and why ZJP demonstrates therapeutic effect on CNG, a clinical trial was conducted. Metabolomics was used to explore its deep mechanism.

**Methods:** A total of 14 patients with CNG were recruited from October 2020 to March 2021 (ChiCTR2000040549). The endoscopy and histopathological changes were evaluated as efficacy. Serum samples were prepared and detected by performing widely targeted metabolome using UPLC. Multivariate statistical analysis was conducted to identify potential differential metabolites and signaling pathways. Last, the signal-related inflammatory factors containing COX-2, IL-4, and IL-17 were confirmed via immunohistochemical staining and enzyme-linked immunosorbent assay.

**Results:** ZJP was able to alleviate several indexes of mucosal injury under endoscopy and histology. Erosion and bile reflux, but not red plaques and hemorrhage, were downregulated by ZJP. In addition, it could remarkably alleviate active chronic inflammation. A total of 14 potential metabolites, namely, hypoxanthine, adipic acid, D-ribono-1,4-lactone, L-sepiapterin, imidazoleacetic acid, sebacate, ADP-ribose, 4-hydroxybenzyl alcohol, 11,12-EET, 15-OxoETE, 12-OxoETE, (±)8-HETE, glycyrrhizinate, and DL-aminopimelic acid, were discriminated by metabolomics. Moreover, certain amino acid metabolism got significance during the disease progress and treatment. The related inflammatory factors including COX-2, IL-4, and IL-17 were inhibited by ZJP in both mucosa and serum.

**Conclusion:** All these results indicated that ZJP partially acts as an inflammatory suppressor to regulate comprehensive metabolism disorders. This might be an important mechanism of ZJP in the treatment of CNG.

## Introduction

Chronic nonatrophic gastritis (CNG) is mainly a common digestive system disease characterized by the infiltration of chronic inflammatory cells without the atrophic mucosal layer. It has always been overlooked owing to the lack of a specific symptom in the past few decades. Even so, accumulative evidence indicates that CNG is the preliminary stage of chronic gastritis and could gradually progress into atrophic gastritis or even gastric carcinoma (Lin et al., 2019). Its discomfort accompanied with distention, belching, acid regurgitation, and nausea also intensifies the afflictive experience (Zhang et al., 2014). Both of these two aspects have aroused more and more attention during CNG progress. The specific agent designed for alleviating both the symptom and the pathogenesis is therefore urgently needed for CNG patients. Zuojin pill (ZJP), a well-known formula from traditional Chinese medicine (TCM), has long been used for chronic gastritis treatment. It was initially created by Danxi Zhu, as recorded in his *Danxi’s Experiential Therapy*, and has served as a digestive system disease agent since the 15th century. Our previous preclinical research indicated that ZJP exerted therapeutic effects on *H. pylori*–induced chronic atrophic gastritis by downregulating the JMJD2B/COX-2/VEGF axis and HMGB1/NF-κB signaling pathway (Wen et al., 2022). N-methyl-N′-nitro-N-nitrosoguanidine–induced chronic atrophic gastritis could also be ameliorated by ZJP through the TGF-β1/PI3K/Akt axis (Tong et al., 2021). In addition, its constituents *Rhizoma coptidis* and *Fructus evodiae* demonstrated explicit biological activity in rat models (Wang et al., 2012; Luo et al., 2021). However, clinical evidence for this efficacy has never been verified. Is ZJP effective for CNG during clinical application? What is the mechanism related to its efficacy? These are inevitable questions for the development of ZJP.

Metabolomics, as an advanced technology and analytical approach, has not been so successful in clarifying the mechanism of ZJP till now. It integrates the network analytic technology and provides more specific insight into biological activities at the serum, tissue, or molecular level. There are more and more studies focusing on metabolomics to shed light on the extensive regulation of herbal medicine in the current time. A recent study by Chen’s group revealed the mechanism of clinical efficacy on compound Shenlu granule for aplastic anemia via metabolomics (Feng et al., 2021). The result indicated that aminoacyl-tRNA biosynthesis and glycerophospholipid metabolism were the key regulatory pathways to restore the pathological process. Another similar study investigated the clinical characteristic of another classical traditional Chinese formula Taohong siwu decoction on coronary heart disease. A total of 27 metabolites mainly including glycerophosphocholine, 8,9-DiHETrE, and 5′-methylthioadenosine were identified. Moreover, the downregulation of glycerophospholipid metabolism and arachidonic acid metabolism was thought to be the crucial signaling pathway for the efficacy (Tao et al., 2020). The related scientific research that explores the how and why questions in TCM was also applied with a randomized controlled trial and metabolomics. Yu’s team found that GeGen decoction could remarkably alleviate primary dysmenorrhea. The mechanism was characterized by network manner. The regulation of pituitary–hypothalamic–ovarian hormones and interfering with metabolic change played a key role during this process (Chai et al., 2020). All these studies provide the possibility of the technical application of ZJP for CNG.

In order to understand how and why ZJP demonstrates therapeutic effect on CNG, a clinical trial was conducted. Metabolomics was used to explore its deep mechanism. We state that this study can not only provide the scientific basis of ZJP in clinical application but also construct a significative method for TCM exploration.

## Methods and Design

### Recruitment and Study Design

Patients with CNG were recruited from October 2020 to March 2021 at The Fifth Medical Center of PLA General Hospital (ChiCTR2000040549). The protocol was approved by the Ethics Committee at The Fifth Medical Center of PLA General Hospital (approval number: 2019087D). The whole procedures and purpose relevant to the trial were clarified to all the patients. Participants with CNG were diagnosed according to the Chinese Consensus on Chronic Gastritis (Shanghai, 2017). The inclusion criteria were as follows: 1) diagnosis of CNG according to the Chinese Consensus on Chronic Gastritis, 2) aged between 35 and 70 years, and 3) voluntary provision of written informed consent. The exclusion criteria were as follows: 1) patients with gastric related surgery; 2) patients with atrophic changes, intestinal metaplasia, or suspected malignant changes in the gastric mucosa; 3) patients with comorbidities of other system diseases in the heart, liver, lung, kidney, or blood system; 4) female patients who were going to having a baby, pregnant, or lactating; 5) patients with life-threatening illness such as tumor; 6) patients who were received other kinds of medicine in the last 2 weeks before recruitment; and 7) patients judged as inappropriate to be enrolled in the trial by investigators. The rejection criteria were as follows: 1) misdiagnosis, 2) patients who did not follow the drug dosage taking; and 3) patients who could not complete the follow-up.

The recruited 14 patients were receiving ZJP for 2-week treatment. ZJP was available from the Department of Pharmacy at The Fifth Medical Center of PLA General Hospital with the dosage of 6.0 g/day. It was made with the root of *Coptis chinensis* Franch. and the fruit of *Euodia ruticarpa* (A. Juss.) Benth., which were weighed and combined in a 6:1 ratio (Beijing Tcmages Pharmaceutical Corporation Limited, batch numbers: 20010241 and 20017291 respectively). During the 2-week hospitalized treatment, the healthy lifestyle, which includes eating a light diet, regularly working and resting, properly exercising, not smoking and drinking alcohol, and avoiding spicy food, was required for patients.

### Outcome Measures

The standard was according to Chinese Consensus on Chronic Gastritis. The score of endoscopy evaluation, involving red plaques, erosion, hemorrhage, and bile reflux, was compared between groups and treatment. The grade of histopathological changes containing chronic inflammation and active chronic inflammation was also evaluated. Chronic inflammation refers to the severity of chronic inflammatory cells localizing in the mucosa, whereas active chronic inflammation is assessing the infiltration of neutrophils with abscess in activity (Zhang et al., 2014).

### Serum Sample Collection and Preparation

Five milliliters of serum sample from 2-week treatment in each group and 10 healthy volunteers was collected and then stored in a freezer at −80°C. Once the analysis was performed, the serum was thawed, and subsequently coagulated for 30 min at 4°C and centrifuged at 3,000×g for 15 min. Then, 300 μl of prechilled 20% acetonitrile methanol was added into 50 μl serum. The mixture was centrifuged at 12,000 r/min for 10 min at 4°C to obtain the supernatant. The supernatant was then dried before storage at −20°C for 20 min, and 180 μl of the sample for analysis was further centrifuged at 12,000 r/min for 3 min at 4°C. A pooled QC sample solution was prepared by combining equal volumes of the serum from each sample. Once one QC sample ended, the instrumental stability was controlled during the batch process.

### T3 UPLC Conditions and ESI-quadrupole-linear ion trap mass spectrometer-MS/MS

Samples were detected by performing widely targeted metabolome using Waters ACQUITY UPLC combined with the Xevo TQ-S Micro mass spectrometer. Waters ACQUITY UPLC HSS T3 C18 (1.8 µm, 2.1 mm × 100 mm) was first used for separation. Ultrapure water with 0.1% formic acid was used as Solvent A, whereas acetonitrile with 0.1% formic acid was used as Solvent B. The gradient program was as follows: 95:5 V/V at 0 min, 10:90 V/V at 11.0 min, 10:90 V/V at 12.0 min, 95:5 V/V at 12.1 min, and 95:5 V/V at 14.0 min.

LIT and triple quadrupole (QQQ) scans were acquired on a triple quadrupole-linear ion trap mass spectrometer (QTRAP), QTRAP^®^ LC-MS/MS System, equipped with an ESI Turbo Ion-Spray interface, operating in positive and negative ion modes and controlled using Analyst 1.6.3 software (Sciex). The ESI source operation parameters were as follows: source temperature 500°C; ion-spray voltage (IS) 5,500 V (positive), −4,500 V (negative); ion source gas I (GSI), gas II (GSII), curtain gas (CUR) were set at 55, 60, and 25.0 psi, respectively; the collision gas (CAD) was high. Instrument tuning and mass calibration were performed with 10 and 100 μmol/L polypropylene glycol solutions in QQQ and LIT modes, respectively. A specific set of MRM transitions were monitored for each period according to the metabolites eluted within this period.

### Immunohistochemical Staining

The expression of COX-2, IL-4, and IL-17 levels in patients’ gastric tissue was measured by immunohistochemical staining. The procedure was performed as follows. The tissue was mixed with 4% paraformaldehyde and embedded in paraffin. Anti-COX-2 antibody (ab179800, Abcam, dilution: 1:100), anti-IL-4 antibody (ab239508, Abcam, dilution: 1:100), and anti-IL-17 antibody (ab79056, Abcam, dilution: 1:100) were used. The images were photographed on Nikon Eclipse Ni-U microscope plus Imaging Software NIS-Elements 4.0 (Nikon, Japan).

### Key Inflammatory Factors Assessment Based on Enzyme-Linked Immunosorbent Assay

The expression of COX-2 (E-EL-H1846c, Elabscience), IL-4 (E-EL-H0101c, Elabscience), and IL-17a (EK117/2-02, MULTISCIENCES) levels in patients’ serum was detected using ELISA. All the serum samples were diluted 10 times. Then 100 μl each dilution of standard, blank, and sample was added into 96-well plates. After adding biotinylated detection Ab working solution, the wash buffer was used for washing. Moreover, HRP conjugate working solution and substrate reagent were then added for reaction. The stop solution was finally applied for assessment.

### Data Processing and Statistical Analysis

The Statistical Package for the Social Sciences version 21.0 was used for clinical efficacy statistical analysis. Student’s t-test was performed to explore the difference between two groups with baseline and efficacy indexes. Values are presented as mean ± standard deviation. *p* < 0.05 and *p <* 0.01 were considered statistically significant and remarkably significant, respectively.

Multivariate statistical analysis was performed using SIMCA-P 14.0 software. Unsupervised principal component analysis (PCA) was used to initially evaluate the quality, homogeneity, and outlier identification of the dataset. The hierarchical cluster analysis (HCA) results of samples and metabolites are presented as heatmaps with dendrograms. Metabolites regulated significantly between groups were determined by VIP ≥ 1 and absolute Log2FC (fold change) ≥ 1. VIP values were extracted from the orthogonal partial least-squares discrimination analysis (OPLS-DA) result, which also contains score plots and permutation plots, generated using the R package MetaboAnalystR. The data were log-transformed (log2) and mean centered before OPLS-DA. Identified metabolites were annotated using the KEGG Compound database (http://www.kegg.jp/kegg/compound/), and annotated metabolites were then mapped to the KEGG Pathway database (http://www.kegg.jp/kegg/pathway.html). Pathways enriched significantly were identified using a hypergeometric test’s *p*-value for a given list of metabolites.

## Results

### Comparison of Outcome Measures

Fourteen patients were determined to be eligible *via* endoscopy evaluation. In addition, the 14 patients, involving 8 men and 6 women, were 51.43 ± 10.68 years old. There were no withdrawn patients after ZJP treatment. ZJP could remarkably alleviate erosion, hemorrhage, bile reflux, and active chronic inflammation score after treatment (*p* < 0.0001, *p* = 0.001). In addition, it could not significantly decrease the red plaques (*p* = 0.205). In addition, it showed an obvious decreasing trend for chronic inflammation (*p* = 0.057) (Table 1). There was no serious report about adverse events from the patients. Just one patient was suspected with slight constipation during the beginning 3 days.

**TABLE 1:** Comparison of endoscopy and histology evaluation before or after Zuojin pill (ZJP) treatment.

Outcome Measures	Before ZJP (n = 14)	After ZJP (n = 14)	*p*-Value
Red plaques	2.07 ± 0.83	1.57 ± 1.09	0.205
Erosion	1.86 ± 0.66	1.07 ± 0.62	<0.0001
Hemorrhage	1.78 ± 0.80	0.50 ± 0.76	<0.0001
Bile reflux	1.86 ± 0.86	0.57 ± 0.76	<0.0001
Chronic inflammation	1.71 ± 0.73	1.07 ± 0.92	0.057
Active chronic inflammation	2.14 ± 0.66	0.93 ± 0.83	0.001

Notes: *p* < 0.05, significance; *p* < 0.01, remarkable significance.

### Serum Metabolites Identification

**FIGURE 1:**
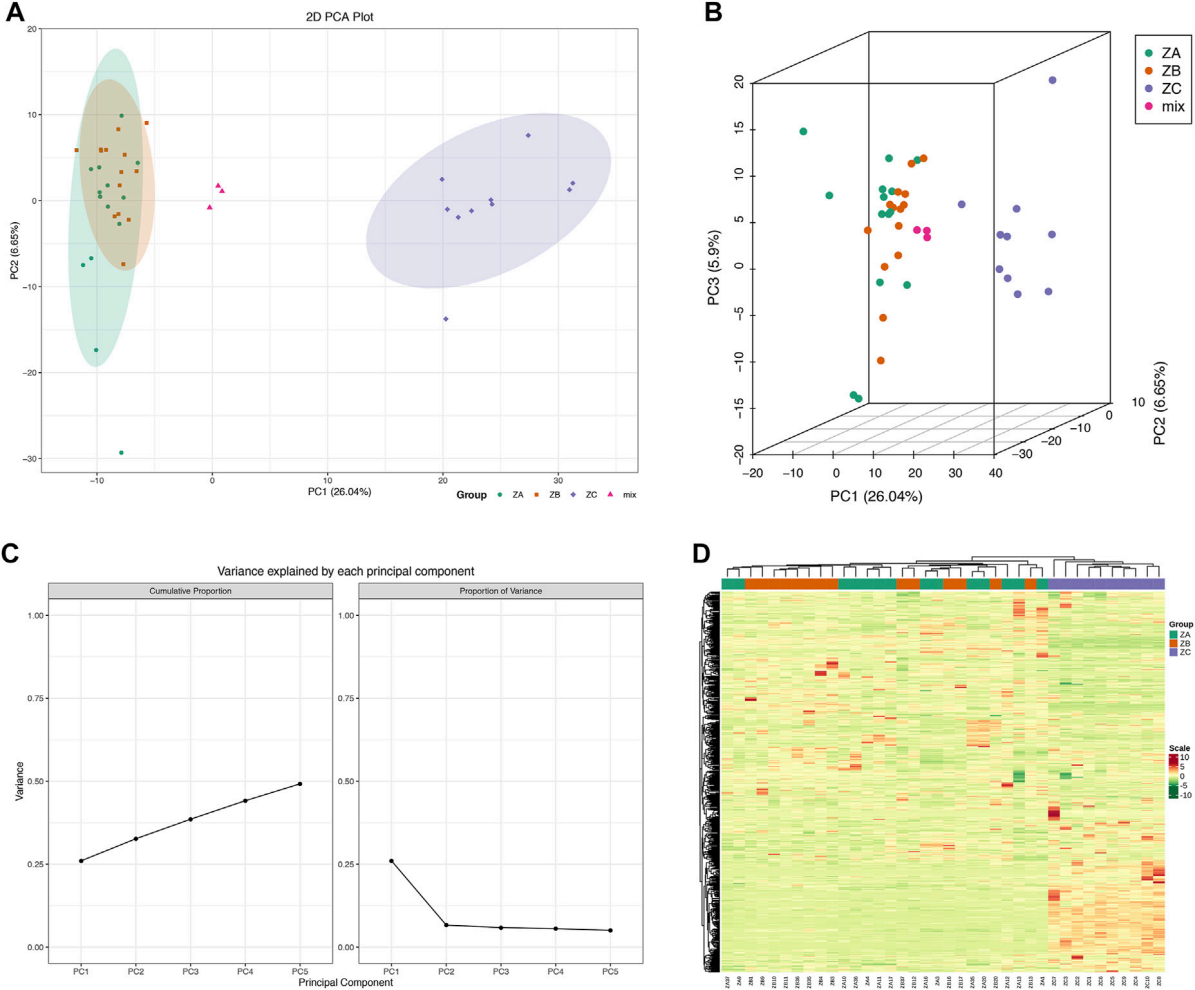
Serum metabolites identification based on principal component analysis (PCA) and hierarchical cluster analysis (HCA). (**(A)** Serum metabolites plot based on PCA in two dimension; **(B)** serum metabolites plot based on PCA in three dimension; **(C)** PCA with multiple variance characteristic; **(D)** serum metabolites heatmap based on HCA; ZA, Zuojin pill (ZJP) after treatment; ZB, ZJP before treatment; ZC, ZJP control as healthy people; mix: quality control).

A total of 789 peaks were detected from 38 serum samples. Pooled quality control samples demonstrated good repeatability and coefficient of variation (Supplementary Figure S1A–D), which indicated that stable data could be acquired from the samples. PCA was initially used as an unsupervised statistical method to study differences in the metabolome before and after treatment as well as among healthy people. However, the clustering could significantly figure out the healthy and patients’ samples. It was not able to distinguish the three groups in PCA although with multiple variances (Figures 1A–C). The heatmap of HCA further indicated that the metabolites of patients’ samples were different (Figure 1D). However, it also could not distinguish the difference between ZJP after treatment (ZA) and ZJP before treatment (ZB).

**FIGURE 2:**
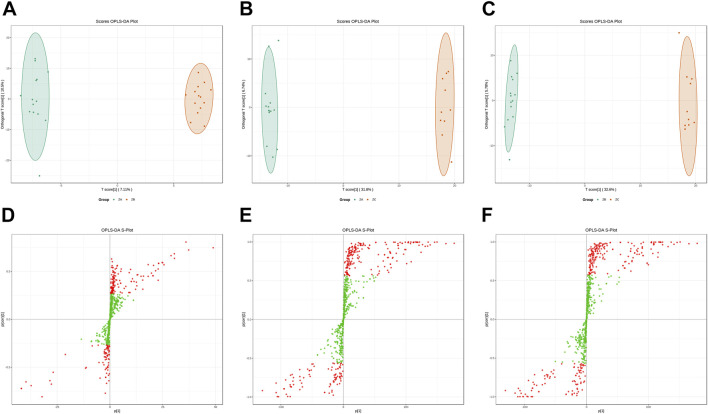
Serum metabolites identification based on the orthogonal partial least-squares discrimination analysis (OPLS-DA). (**(A)** Serum metabolites score plot based on OPLS-DA between ZA and ZB; **(B)** serum metabolites score plot based on OPLS-DA between ZA and ZC; **(C)** serum metabolites score plot based on OPLS-DA between ZB and ZC; **(D)** S-plot between ZA and ZB; **(E)** S-plot between ZA and ZC; **(F)** S-plot between ZB and ZC; ZA, ZJP after treatment; ZB, ZJP before treatment; ZC, ZJP control as healthy people).

In order to discriminate the distinctive metabolites, multivariate statistical analysis was further applied. OPLS-DA was then applied to differentiate the metabolomics patterns and identify potential changes in the biomarker level. The characteristic of ZA, ZB, and ZC could be discriminated from each other in the OPLS-DA score plot (Figures 2A–C). The R^2^X, R^2^Y, and Q^2^ of OPLS-DA to clarify ZA and ZB were 0.329, 0.994, and 0.657, respectively. The R^2^X, R^2^Y, and Q^2^ of OPLS-DA to clarify ZA and ZJP control (healthy people as ZC) were 0.386, 0.998, and 0.981, respectively. Moreover, the R^2^X, R^2^Y, and Q^2^ of OPLS-DA to clarify ZB and ZC were 0.384, 0.999, and 0.983, respectively. These data indicated that the models are of good fitness and predictability. In addition, the S-plot indicated the potential biomarkers from OPLS-DA when VIP ≥ 1 with red dots (Figures 2D–F).

**FIGURE 3:**
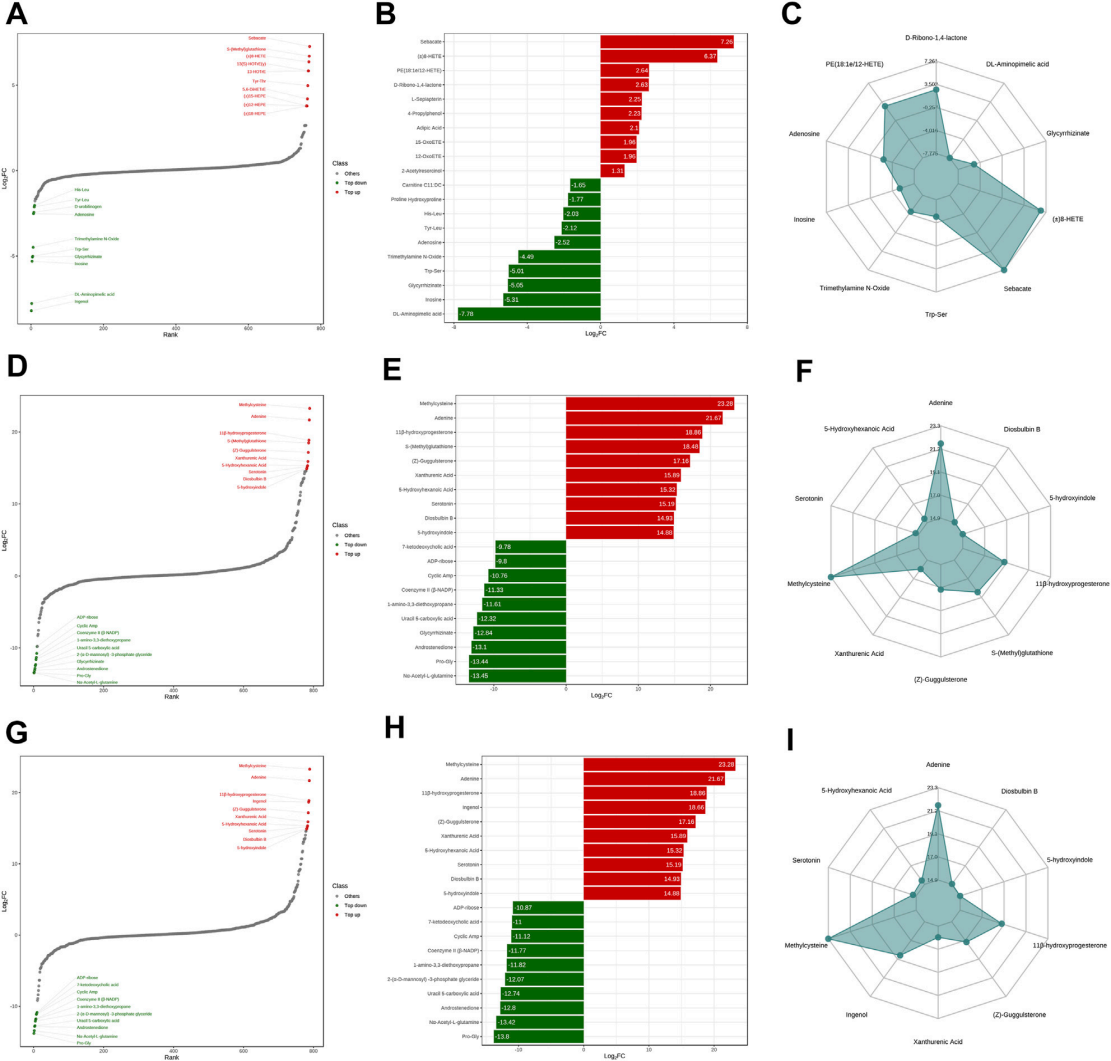
Identified differential metabolites characteristic. (**(A–C)** Dynamic distribution of upregulated and downregulated metabolites between ZA and ZB; **(D–F)** dynamic distribution of upregulated and downregulated metabolites between ZA and ZC; **(G–I)** dynamic distribution of upregulated and downregulated metabolites between ZB and ZC; 10 most upregulated metabolites were with red dots/bar and 10 most downregulated metabolites were with green dots/bar; Radar map selectively demonstrates 10 representative metabolites with change score).

At last, a total of 39, 240, and 248 metabolites were respectively identified between ZA and ZB, ZA and ZC, and ZB and ZC (Supplementary Table S1). Based on the fold change values, the top 10 upregulated metabolites and downregulated metabolites of ZA and ZB were respectively sebacate, S-(methyl)glutathione, (±)8-HETE, 13(S)-HOTrE(γ), 13-HOTrE, Tyr-Thr, 5,6-DiHETrE, (±)15-HEPE, (±)12-HEPE, and (±)18-HEPE and His-Leu, Tyr-Leu, D-urobilinogen, adenosine, trimethylamine N-oxide, Trp–Ser, glycyrrhizinate, inosine, DL-aminopimelic acid, and ingenol (Figures 3A–C). As for ZA and ZC, methylcysteine, adenine, 11β-hydroxyprogesterone, S-(methyl)glutathione, (Z)-guggulsterone, xanthurenic acid, 5-hydroxyhexanoic acid, serotonin, diosbulbin B, 5-hydroxyindole were regulated, and 7-ketodeoxycholic acid, ADP-ribose, cyclic AMP, coenzyme II (β-NADP), 1-amino-3,3-diethoxypropane, uracil 5-carboxylic acid, glycyrrhizinate, androstenedione, Pro–Gly, and Nα-acetyl-l-glutamine were downregulated (Figures 3D–F). Methylcysteine, adenine, 11β-hydroxyprogesterone, ingenol, (Z)-guggulsterone, xanthurenic acid, 5-hydroxyhexanoic acid, serotonin, diosbulbin B, and 5-hydroxyindole were upregulated, whereas ADP-ribose, 7-ketodeoxycholic acid, cyclic AMP, coenzyme II (β-NADP), 1-amino-3,3-diethoxypropane, 2-(α-d-mannosyl)-3-phosphate glyceride, uracil 5-carboxylic acid, androstenedione, Nα-acetyl-l-glutamine, and Pro–Gly were downregulated in the ZB and ZC comparison (Figures 3G–I). The Venn figure was further applied in order to get the precise difference among the three groups. Fourteen potential metabolites, namely, hypoxanthine, adipic acid, D-ribono-1,4-lactone, L-sepiapterin, imidazoleacetic acid, sebacate, ADP-ribose, 4-hydroxybenzyl alcohol, 11,12-EET, 15-OxoETE, 12-OxoETE, (±)8-HETE, glycyrrhizinate, and DL-aminopimelic acid, were identified (Table 2, Supplementary Figure S2). From the result, we could see that most of the metabolites belonged to the classes of nucleotide and its metabolomics, organic acid and its derivatives, and oxidized lipids.

**TABLE 2:** Differential metabolites among ZJP before or after treatment and healthy people.

Number	Formula	Compounds	Classes	ZA	ZB	ZC	ZA/ZB	ZA/ZC	ZB/ZC
1	C5H4N4O	Hypoxanthine	Nucleotide and Its metabolomics	4.36E+07 ± 3.64E+07	1.95E+07 ± 8.27E+06	3.08E+08 ± 5.22E+07	up	down	down
2	C6H10O4	Adipic Acid	Organic acid And Its derivatives	9.73E+03 ± 1.94E+04	4.18E+04 ± 1.42E+04	2.39E+05 ± 6.68E+04	down	down	down
3	C5H8O5	D-Ribono-1,4-lactone	Sugar acids	1.73E+03 ± 6.44E+03	1.07E+04 ± 1.83E+04	8.97E+05 ± 1.23E+05	down	down	down
4	C9H11N5O3	L-Sepiapterin	Pteridines and derivatives	2.53E+03 ± 9.43E+03	1.20E+04 ± 1.84E+04	3.59E+04 ± 1.33E+04	down	down	down
5	C5H6N2O2	Imidazoleacetic acid	Indole and Its derivatives	1.38E+05 ± 1.19E+05	2.80E+05 ± 1.20E+05	1.68E+04 ± 5.30E+04	down	up	up
6	C10H18O4	Sebacate	Organic acid And Its derivatives	9.00E+00 ± 0.00	1.38E+03 ± 2.79E+03	1.29E+04 ± 5.39E+03	down	down	down
7	C15H23N5O14P2	ADP-ribose	Nucleotide and Its metabolomics	8.04E+03 ± 9.00E+03	1.68E+04 ± 4.93E+03	9.00E+00 ± 0.00	down	up	up
8	C7H8O2	4-Hydroxybenzyl alcohol	Benzene and substituted derivatives	1.02E+05 ± 7.79E+04	5.04E+04 ± 1.60E+04	2.25E+04 ± 6.14E+03	up	up	up
9	C20H32O3	11,12-EET	Oxidized lipids	6.77E+03 ± 5.72E+03	1.39E+04 ± 1.56E+04	2.71E+05 ± 4.09E+05	down	down	down
10	C20H30O3	15-OxoETE	Oxidized lipids	1.07E+03 ± 2.84E+03	4.16E+03 ± 7.83E+03	3.26E+04 ± 6.17E+04	down	down	down
11	C20H30O3	12-OxoETE	Oxidized lipids	1.07E+03 ± 2.84E+03	4.16E+03 ± 7.83E+03	3.26E+04 ± 6.17E+04	down	down	down
12	C20H32O3	(±)8-HETE	Oxidized lipids	9.00E+00 ± 0.00	7.42E+02 ± 2.05E+03	5.51E+04 ± 3.04E+04	down	down	down
13	C42H62O16	Glycyrrhizinate	Organic acid And Its derivatives	1.14E+07 ± 2.88E+07	3.42E+05 ± 1.16E+06	1.55E+03 ± 2.93E+03	up	up	up
14	C7H13NO4	DL-Aminopimelic acid	Organic acid And Its derivatives	1.97E+03 ± 4.99E+03	9.00E+00 ± 0.00	1.51E+05 ± 5.71E+04	up	down	down

Notes: ZA, ZJP after treatment; ZB, ZJP before treatment; ZC, ZJP control as healthy people.

### Metabolites Pathways Analysis

**FIGURE 4:**
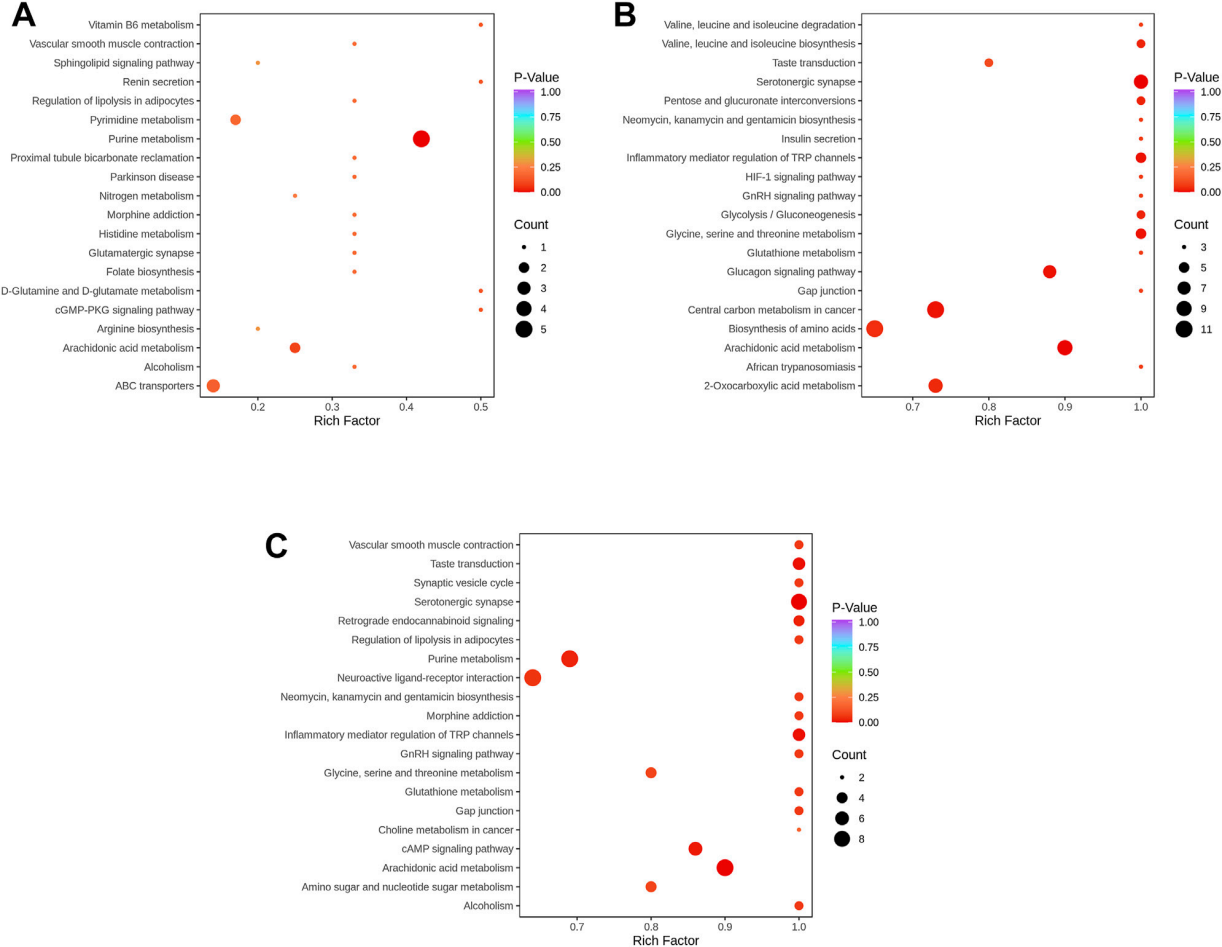
KEGG Enrichment analysis of differential metabolites. (**(A)** ZA vs. ZB; **(B)** ZA vs. ZC; **(C)** ZB vs. ZC; the color of the dot is *p*-value. The redder it is, the more significant the enrichment is. The size of the dot represents the number of differential metabolites in pathway enriched).

Detailed analyses of pathways were performed by both KEGG and metabolic set enrichment analysis (MSEA). The metabolic signals in KEGG revealed that pyrimidine metabolism, purine metabolism, and ABC transporters were responsible for the alleviation of CNG after ZJP treatment (Figure 4A). When compared with those in healthy samples, serotonergic synapse, central carbon metabolism in cancer, and biosynthesis of amino acids were the main regulated signals during ZJP treatment (Figure 4B). Moreover, purine metabolism, neuroactive ligand–receptor interaction, inflammatory mediator regulation of TRP channels, synaptic vesicle cycle, arachidonic acid metabolism, and cAMP signaling pathway were regulated compared with those in CNG samples and healthy samples (Figure 4C).

**FIGURE 5:**
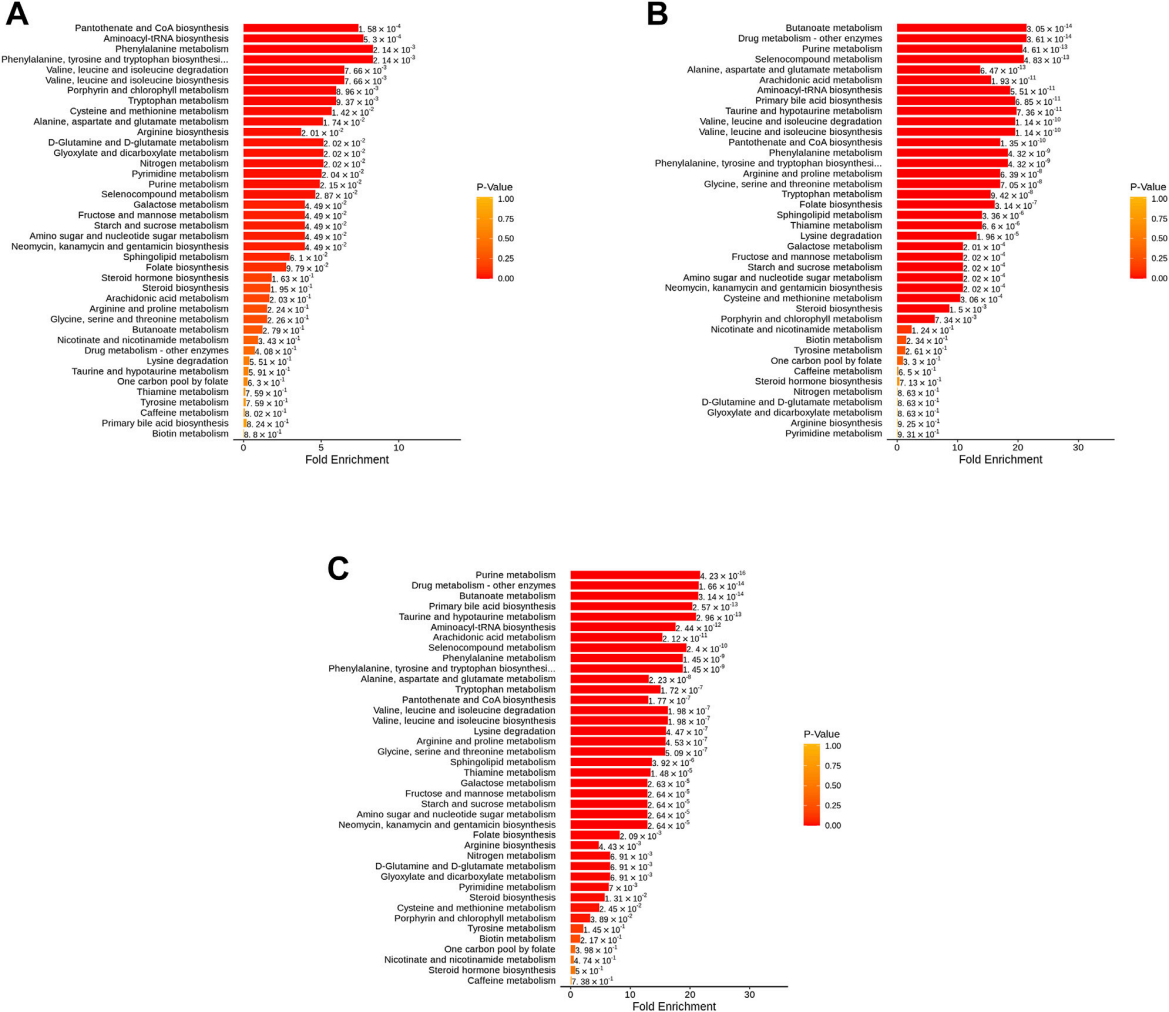
MSEA of differential metabolites. (**(A)** ZA vs. ZB; **(B)** ZA vs. ZC; **(C)** ZB vs. ZC; The color of the dot represents *p*-value).

MSEA does not need to specify a clear threshold of differential metabolites. It aims to set a series of metabolic sets, and each metabolic set represents a certain biological function. The enriched data could statistically find the metabolic sets with significant differences. The result indicated that pantothenate and CoA biosynthesis, aminoacyl-tRNA biosynthesis, phenylalanine metabolism, and phenylalanine, tyrosine, and tryptophan biosynthesis have specific importance between ZA and ZB (Figure 5A, Supplementary Table S2). Butanoate metabolism, drug metabolism (other enzymes), purine metabolism, and selenocompound metabolism were responsible for the difference signals of ZA and ZC (Figure 5B, Supplementary Table S3). Moreover, Purine metabolism, drug metabolism (other enzymes), butanoate metabolism, and primary bile acid biosynthesis were important in ZB and ZC (Figure 5C, Supplementary Table S4). From the result, we could see that the amino acid metabolism demonstrated remarkable significance during the disease progression and treatment.

### The Confirmation of Possible Effectors

**FIGURE 6:**
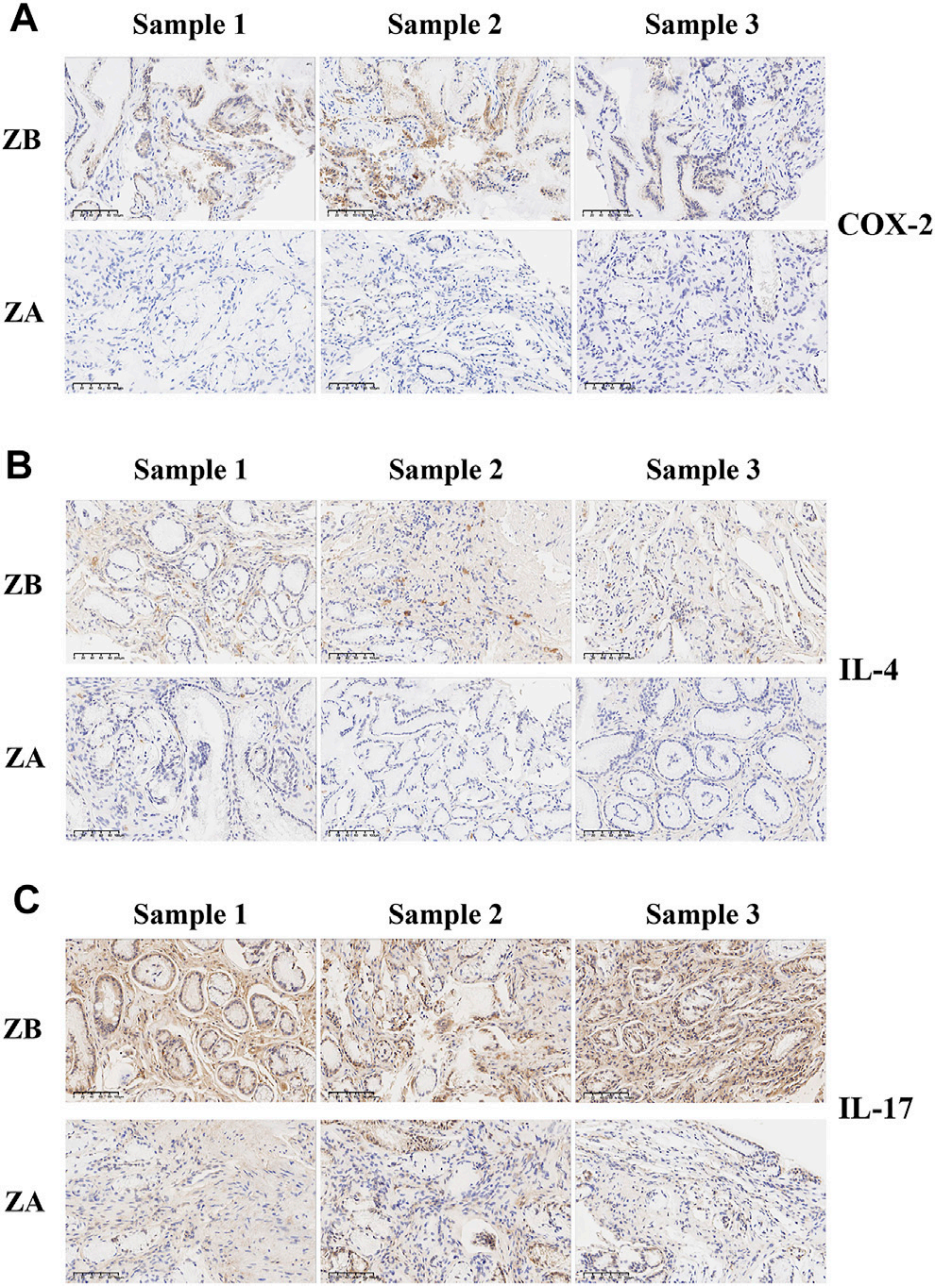
Immunohistochemical staining of inflammatory factors before or after ZJP treatment in gastric mucosa. (**(A)** Expression of COX-2 in patients’ gastric mucosa; **(B)** expression of IL-4 in patients’ gastric mucosa; **(C)** expression of IL-17 in patients’ gastric mucosa; ZA, ZJP after treatment; ZB, ZJP before treatment).

**FIGURE 7:**
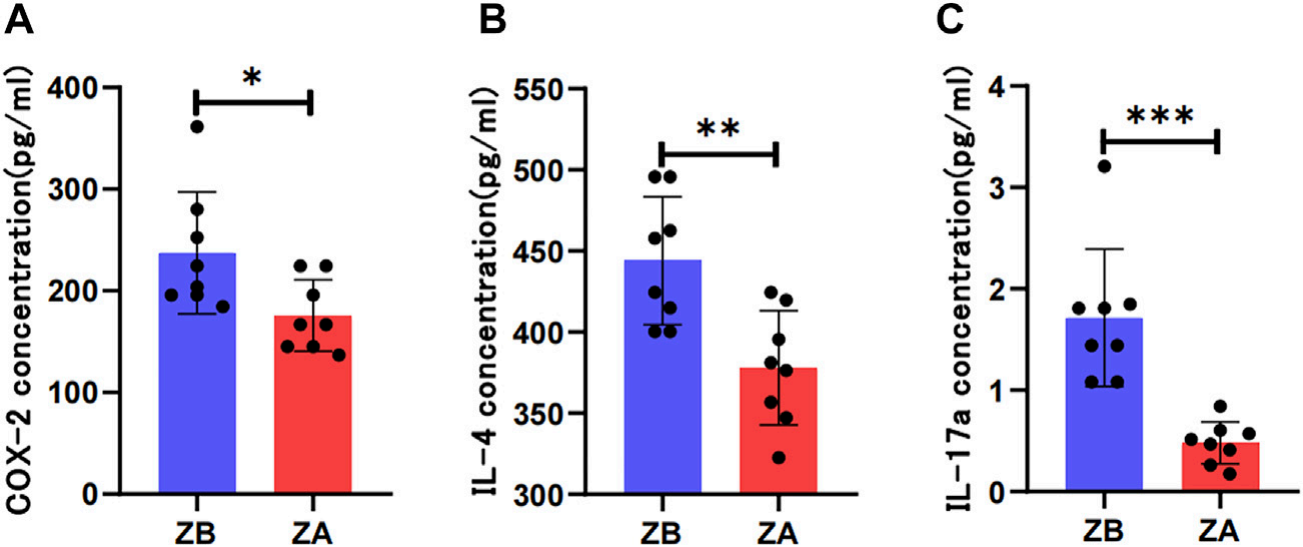
ELISA detection of inflammatory factors before or after ZJP treatment in serum. (**(A)** Expression of COX-2 in patients’ serum; **(B)** expression of IL-4 in patients’ serum; **(C)** expression of IL-17a in patients’ serum; ZA, ZJP after treatment; ZB, ZJP before treatment).

Metabolite pathway analysis demonstrated that the regulation of systematic amino acid metabolism was involved in the efficacy of ZJP. Most recent studies have suggested that the inflammatory status could affect amino acid metabolism in various diseases. Moreover, our previous preclinical research also showed that ZJP could alleviate the metabolism and inflammatory status of chronic gastritis. Thus, the essential inflammatory factors of chronic gastritis were detected in this study. The result indicated that ZJP could downregulate the proinflammatory level of COX-2, IL-4, and IL-17 in patients’ gastric mucosa based on immunohistochemical staining (Figures 6A–C). In addition, the same result was also confirmed in patients’ serum with the remarkable decrease of COX-2, IL-4, and IL-17a (Figures 7A–C).

## Discussion

In the most recent years, network regulation has been thought as an essential characteristic of pathology in complex disease. The concept of combination medication is therefore believed to be a crucial method for disease treatment (Li et al., 2020; Wu et al., 2021; Nogales et al., 2022). In addition, this characteristic has long been presented since the construction of TCM and lasted at least over 2,000 years. More and more traditional Chinese formulas have been widely accepted because of the network regulatory mechanism. ZJP is the representative for the treatment of gastrointestinal disease among these formulas. It contains *Rhizoma coptidis* and *Fructus evodiae* in the ratio of 6:1. This research first focused on the clinical efficacy of ZJP on CNG. The result suggested that ZJP was able to alleviate several indexes of mucosal injury under endoscopy and histology. It could downregulate erosion and bile reflux, but not red plaques and hemorrhage, indicating the selectivity of ZJP action. In addition, it could remarkably alleviate active chronic inflammation. This action is critically important in antiinflammation. Therefore, this efficacy coincided with our previous experimental results. In recent studies, *Rhizoma coptidis* was proven to be able to alleviate chronic gastritis. Previous research revealed that palmatine was the main compound to demonstrate the gastric mucosal protective effect. It might ameliorate chronic gastritis via downregulating the ADAM17/EGFR signaling pathway (Chen et al., 2020). In addition, this biological activity was also relevant to regulating the metabolites network involving taurine and hypotaurine metabolism, glycerophospholipid metabolism, and pentose and glucuronate interconversions. Another primary compound, berberine, derived from *Rhizoma coptidis* also demonstrated significant effect on gastritis. The study indicated that berberine was able to suppress the IRF8–IFN-γ signaling axis to inhibit the pathological progress. Apart from *Rhizoma coptidis*, the research on the effects of *Fructus evodiae* on gastritis has also vastly improved (Yang et al., 2020). Rutaecarpine is a quinazolino carboline alkaloid extracted from *Fructus evodiae*. A recent study revealed that it could exert remarkable gastroprotective effect against ethanol-induced mucosal injury. In addition, this effect is associated with inhibiting nuclear translocation of p65 and the downstream signaling factors. The role of the PI3K/AKT signaling pathway is also involved in this process (Ren et al., 2020). Thus, the preclinical result of network regulation might indicate the core function of ZJP for CNG in clinic. Metabolomics should shed light on the exploration of further mechanisms (Liu et al., 2021).

Metabolomics was further conducted in order to investigate the direct mechanism. The result indicated that 14 potential metabolites, namely, hypoxanthine, adipic acid, D-ribono-1,4-lactone, L-sepiapterin, imidazoleacetic acid, sebacate, ADP-ribose, 4-hydroxybenzyl alcohol, 11,12-EET, 15-OxoETE, 12-OxoETE, (±)8-HETE, glycyrrhizinate, and DL-aminopimelic acid, were relevant to the efficacy of ZJP treatment. In addition, these metabolites suggested that certain amino acid metabolism became significant during the disease progress and treatment. Hence, the comprehensive characteristic was revealed in this study. However, there still exists the question of how ZJP could cause the change of amino acid metabolism. In recent years, some studies focused on the close relationship between inflammation and amino acid change in many inflammatory diseases. Findings suggested that inflammatory factors could significantly dysregulate phenylalanine, tyrosine, tryptophan, and some related amino acids and cause metabolism disorders (Lario et al., 2017; Wang et al., 2021; Zhang et al., 2022). For instance, a study found that patients with gastric carcinoma and inflammatory characteristic always demonstrated higher concentrations of phenylacetylglutamine. Phenylacetylglutamine also serves as a microbial metabolite with inflammatory regulation (Chan et al., 2016). Another study also revealed that tryptophan metabolites of the gut microbiota suppress liver inflammation (Krishnan et al., 2019). From all these, we could see that inflammatory regulation was closely related with ZJP treatment. To combine our previous preclinical research, the representative factors including COX-2, IL-4, and IL-17 were selected for detection. The result finally confirmed that ZJP was able to suppress systematic inflammation and thus improve the metabolism to alleviate CNG.

Overall, this research strictly abided by the clinical and metabolomics standard all over the procedure. However, there still exists improvement in two aspects. First of all, a larger sample from multiple centers is needed for further confirmation. Performing this kind of trial can be the only high evidence chain applied in guide of gastritis treatment. Second, the deep mechanism is waiting for investigation accompanied by space–time multiomics tech (Gu et al., 2020). Owing to the current progress, the exploration of a combined method such as metabolomics and proteomics is urgently needed to investigate the possible mechanism and indicate the molecular index for cure.

## Conclusion

In summary, ZJP partially acts as an inflammatory suppressor to regulate comprehensive metabolism disorders. This might be an important mechanism of ZJP in the treatment of CNG.

## Data Availability

The original contributions presented in the study are included in the article/Supplementary Material, and further inquiries can be directed to the corresponding authors.
