# Supplementary material for: Metabolomics Profiles Associated with the Treatment of Zuojin Pill on Patients with Chronic Nonatrophic Gastritis
Source: Front Pharmacol. 2022 Jul 11;13:898680. doi: 10.3389/fphar.2022.898680 (PMC9310101; doi:10.3389/fphar.2022.898680)
Supplement: Supplementary file 1 [file DataSheet1.docx]

**Metabolomics profiles associated with the treatment of Zuojin pill on** **patients with** **chronic non-atrophic** **gastritis**

**Supplementary File**


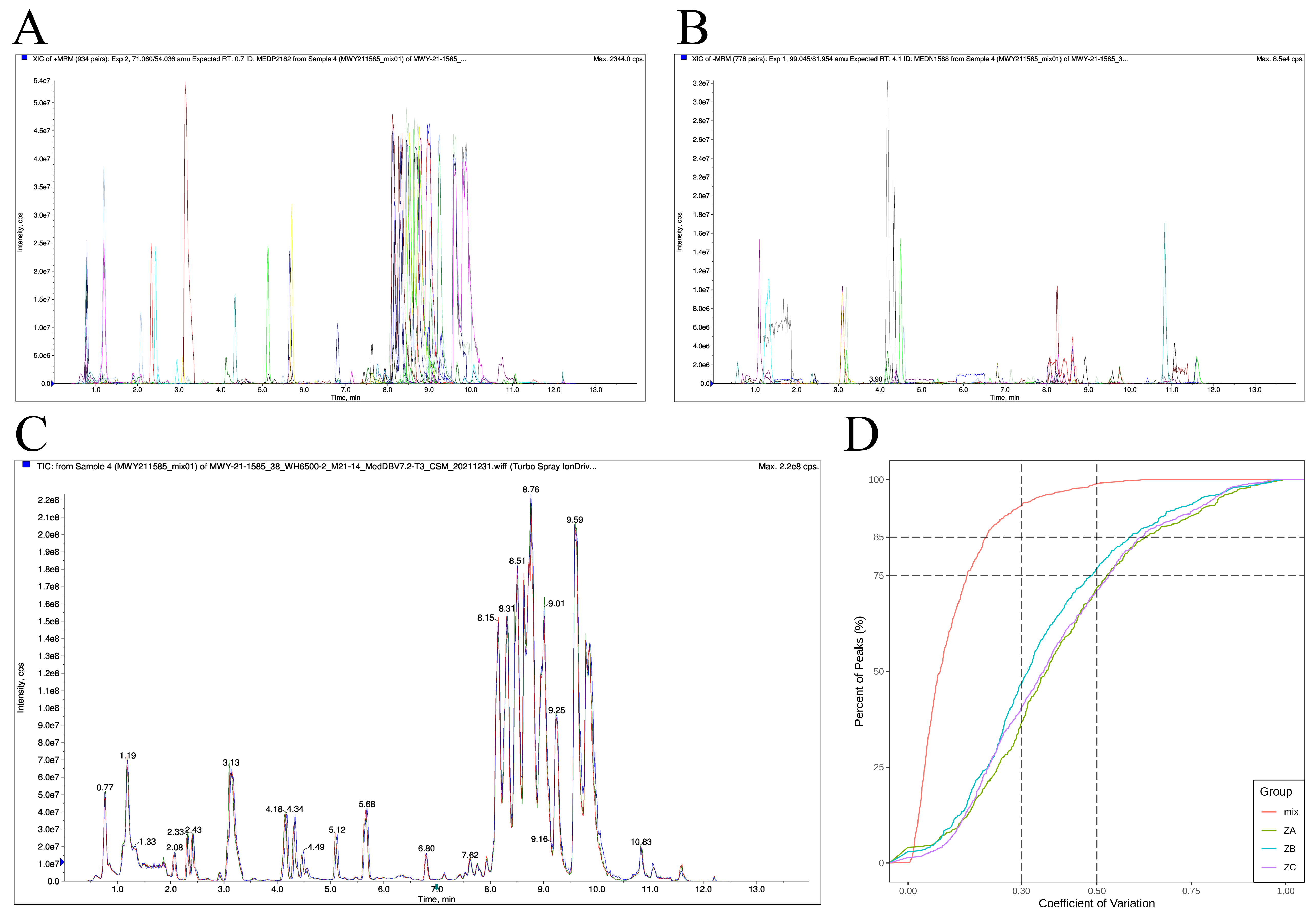


**Figure S1 Total ions current characteristic of serum samples**

(A. Positive total ions current characteristic; B. Negative total ions current characteristic; C. Overlap of total ions current in quality control; D. Coefficient of variation in different samples. ZA: ZJP after treatment; ZB: ZJP before treatment; ZC: ZJP control as healthy people; mix: Quality control)


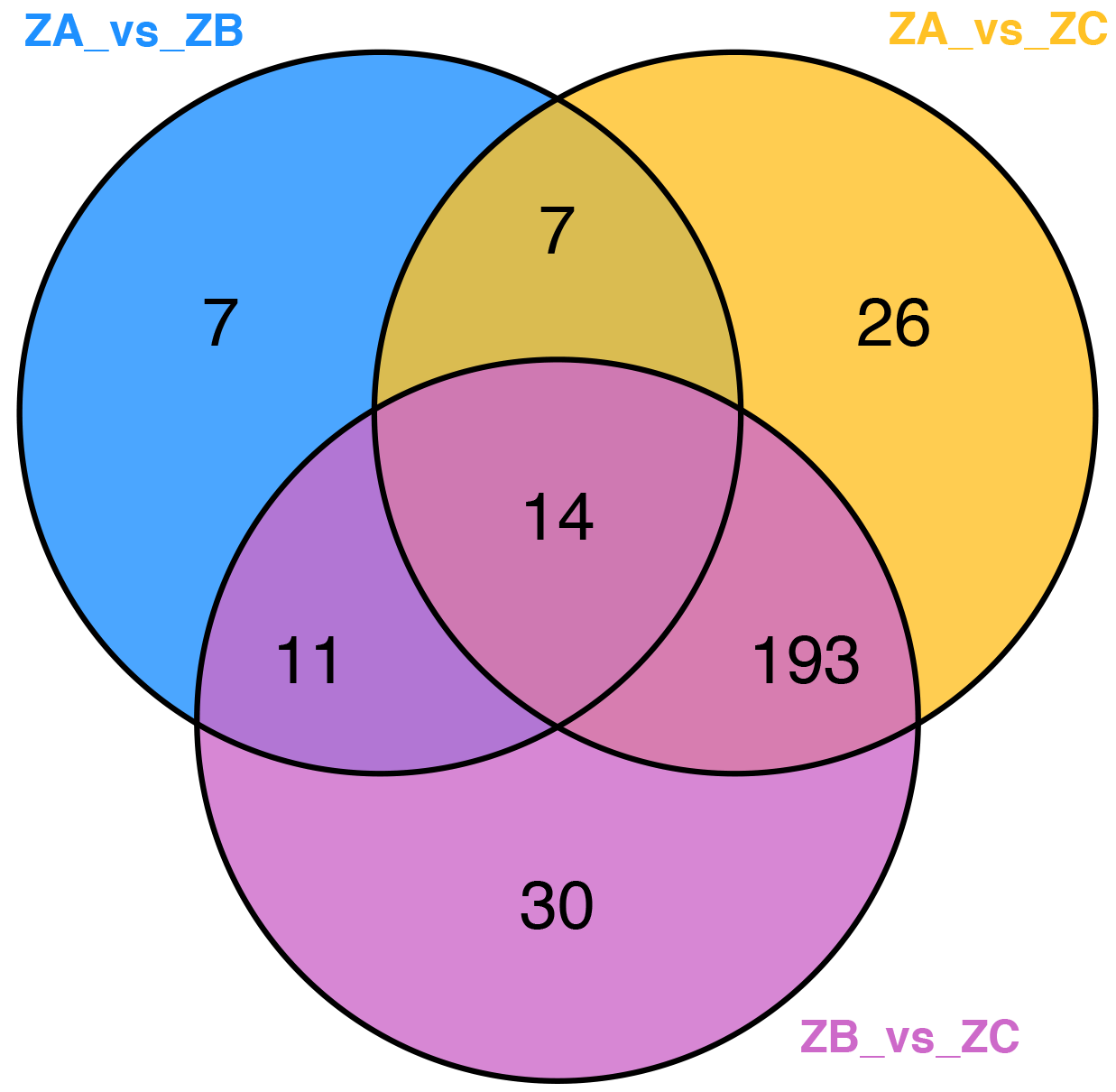


**Figure S2** **Differential metabolites of Venn diagram**

(ZA: ZJP after treatment; ZB: ZJP before treatment; ZC: ZJP control as healthy people)

**Table S1 Identified differential metabolites among serum samples**

| **Group Name** | **Total Significant Metabolites** | **Down-regulated** | **Up-regulated** |
| --- | --- | --- | --- |
| ZA and ZB | 39 | 18 | 21 |
| ZA and ZC | 240 | 71 | 169 |
| ZB and ZC | 248 | 78 | 170 |

Notes: ZA: ZJP after treatment; ZB: ZJP before treatment; ZC: ZJP control as healthy people

**Table S2 Characteristic of MSEA in ZA and ZB**

| **Name** | **Total Compounds** | **Hits** | ***P*-value** | **FDR** | **Fold Enrichment** | **Hit Compounds** |
| --- | --- | --- | --- | --- | --- | --- |
| Pantothenate and CoA biosynthesis | 19 | 2 | 1.58E-04 | 6.32E-03 | 7.40 | Pantetheine,L-Valine |
| Aminoacyl-tRNA biosynthesis | 48 | 9 | 5.30E-04 | 1.06E-02 | 7.73 | L-Phenylalanine,L-Glutamine,L-Methionine,L-Valine,L-Alanine,L-Isoleucine,L-Leucine,L-Tryptophan,10-Formyltetrahydrofolate |
| Phenylalanine metabolism | 10 | 1 | 2.14E-03 | 2.14E-02 | 8.34 | L-Phenylalanine |
| Phenylalanine, tyrosine and tryptophan biosynthesis | 4 | 1 | 2.14E-03 | 2.14E-02 | 8.34 | L-Phenylalanine |
| Valine, leucine and isoleucine degradation | 40 | 3 | 7.66E-03 | 4.68E-02 | 6.50 | L-Valine,L-Isoleucine,L-Leucine |
| Valine, leucine and isoleucine biosynthesis | 8 | 3 | 7.66E-03 | 4.68E-02 | 6.50 | L-Leucine,L-Isoleucine,L-Valine |
| Porphyrin and chlorophyll metabolism | 30 | 2 | 8.96E-03 | 4.68E-02 | 5.97 | Biliverdin,D-Urobilinogen |
| Tryptophan metabolism | 41 | 2 | 9.37E-03 | 4.68E-02 | 5.98 | L-Tryptophan,L-Kynurenine |
| Cysteine and methionine metabolism | 33 | 1 | 1.42E-02 | 5.37E-02 | 5.67 | L-Methionine |
| Alanine, aspartate and glutamate metabolism | 28 | 3 | 1.74E-02 | 5.37E-02 | 5.11 | L-Alanine,L-Glutamine,Carbamoyl phosphate |
| Arginine biosynthesis | 14 | 3 | 2.01E-02 | 5.37E-02 | 3.72 | Carbamoyl phosphate,L-Glutamine,Urea |
| D-Glutamine and D-glutamate metabolism | 6 | 1 | 2.02E-02 | 5.37E-02 | 5.14 | L-Glutamine |
| Glyoxylate and dicarboxylate metabolism | 32 | 1 | 2.02E-02 | 5.37E-02 | 5.14 | L-Glutamine |
| Nitrogen metabolism | 6 | 2 | 2.02E-02 | 5.37E-02 | 5.14 | Carbamoyl phosphate,L-Glutamine |
| Pyrimidine metabolism | 39 | 5 | 2.04E-02 | 5.37E-02 | 5.05 | L-Glutamine,Carbamoyl phosphate,Uridine,Cytidine,Thymine |
| Purine metabolism | 65 | 6 | 2.15E-02 | 5.37E-02 | 4.90 | Xanthine,L-Glutamine,Adenosine,Hypoxanthine,Inosine,Urea |
| Selenocompound metabolism | 20 | 1 | 2.87E-02 | 6.74E-02 | 4.62 | L-Alanine |
| Galactose metabolism | 27 | 4 | 4.49E-02 | 8.17E-02 | 3.94 | Melibiose,D-Glucose,Galactitol,D-Mannose |
| Fructose and mannose metabolism | 20 | 1 | 4.49E-02 | 8.17E-02 | 3.94 | D-Mannose |
| Starch and sucrose metabolism | 18 | 1 | 4.49E-02 | 8.17E-02 | 3.94 | D-Glucose |
| Amino sugar and nucleotide sugar metabolism | 37 | 1 | 4.49E-02 | 8.17E-02 | 3.94 | D-Mannose |
| Neomycin, kanamycin and gentamicin biosynthesis | 2 | 1 | 4.49E-02 | 8.17E-02 | 3.94 | D-Glucose |
| Sphingolipid metabolism | 21 | 4 | 6.10E-02 | 1.06E-01 | 2.99 | Sphingosine 1-phosphate,Sphinganine 1-phosphate,Sphingosine,3-Dehydrosphinganine |
| Folate biosynthesis | 27 | 1 | 9.79E-02 | 1.63E-01 | 2.75 | Sepiapterin |
| Steroid hormone biosynthesis | 85 | 4 | 1.63E-01 | 2.61E-01 | 1.81 | Corticosterone,Cortisol,Androstenedione,18-Hydroxycorticosterone |
| Steroid biosynthesis | 42 | 1 | 1.95E-01 | 2.99E-01 | 1.72 | Calcitriol |
| Arachidonic acid metabolism | 36 | 2 | 2.03E-01 | 3.00E-01 | 1.66 | Leukotriene B4,5,6-DHET |
| Arginine and proline metabolism | 38 | 2 | 2.24E-01 | 3.11E-01 | 1.51 | Creatine,1-Pyrroline-4-hydroxy-2-carboxylate |
| Glycine, serine and threonine metabolism | 33 | 1 | 2.26E-01 | 3.11E-01 | 1.51 | Creatine |
| Butanoate metabolism | 15 | 1 | 2.79E-01 | 3.72E-01 | 1.21 | 2-Hydroxyglutarate |
| Nicotinate and nicotinamide metabolism | 15 | 1 | 3.43E-01 | 4.42E-01 | 0.94 | N1-Methyl-2-pyridone-5-carboxamide |
| Drug metabolism - other enzymes | 39 | 1 | 4.08E-01 | 5.10E-01 | 0.71 | Isonicotinic acid |
| Lysine degradation | 25 | 1 | 5.51E-01 | 6.68E-01 | 0.37 | N6,N6,N6-Trimethyl-L-lysine |
| Taurine and hypotaurine metabolism | 8 | 1 | 5.91E-01 | 6.95E-01 | 0.30 | Taurine |
| One carbon pool by folate | 9 | 1 | 6.30E-01 | 7.20E-01 | 0.24 | 10-Formyltetrahydrofolate |
| Thiamine metabolism | 7 | 1 | 7.59E-01 | 8.21E-01 | 0.10 | Thiamine |
| Tyrosine metabolism | 42 | 2 | 7.59E-01 | 8.21E-01 | 0.15 | Dopaquinone,Thyroxine |
| Caffeine metabolism | 10 | 3 | 8.02E-01 | 8.44E-01 | 0.07 | 1-Methylxanthine,Theobromine,Caffeine |
| Primary bile acid biosynthesis | 46 | 2 | 8.24E-01 | 8.45E-01 | 0.20 | Taurine,Cholic acid |
| Biotin metabolism | 10 | 1 | 8.80E-01 | 8.80E-01 | 0.02 | Biotin |

Notes: *P*<0.05, significance; *P*<0.01, remarkable significance; ZA: ZJP after treatment; ZB: ZJP before treatment

**Table S3 Characteristic of MSEA in ZA and ZC**

| **Name** | **Total Compounds** | **Hits** | ***P*-value** | **FDR** | **Fold Enrichment** | **Hit Compounds** |
| --- | --- | --- | --- | --- | --- | --- |
| Butanoate metabolism | 15 | 1 | 3.05E-14 | 7.21E-13 | 21.41 | 2-Hydroxyglutarate |
| Drug metabolism - other enzymes | 39 | 1 | 3.61E-14 | 7.21E-13 | 21.38 | Isonicotinic acid |
| Purine metabolism | 65 | 7 | 4.61E-13 | 4.83E-12 | 20.70 | Xanthine,L-Glutamine,Adenosine,Hypoxanthine,Inosine,Adenine,Urea |
| Selenocompound metabolism | 20 | 1 | 4.83E-13 | 4.83E-12 | 20.95 | L-Alanine |
| Alanine, aspartate and glutamate metabolism | 28 | 3 | 6.47E-13 | 5.18E-12 | 13.74 | L-Alanine,L-Glutamine,Carbamoyl phosphate |
| Arachidonic acid metabolism | 36 | 3 | 1.93E-11 | 1.29E-10 | 15.52 | Leukotriene B4,Prostaglandin D2,5,6-DHET |
| Aminoacyl-tRNA biosynthesis | 48 | 9 | 5.51E-11 | 3.15E-10 | 18.67 | L-Phenylalanine,L-Glutamine,L-Methionine,L-Valine,L-Alanine,L-Isoleucine,L-Leucine,L-Tryptophan,10-Formyltetrahydrofolate |
| Primary bile acid biosynthesis | 46 | 2 | 6.85E-11 | 3.27E-10 | 19.56 | Taurine,Cholic acid |
| Taurine and hypotaurine metabolism | 8 | 1 | 7.36E-11 | 3.27E-10 | 19.78 | Taurine |
| Valine, leucine and isoleucine degradation | 40 | 3 | 1.14E-10 | 4.15E-10 | 19.54 | L-Valine,L-Isoleucine,L-Leucine |
| Valine, leucine and isoleucine biosynthesis | 8 | 3 | 1.14E-10 | 4.15E-10 | 19.54 | L-Leucine,L-Isoleucine,L-Valine |
| Pantothenate and CoA biosynthesis | 19 | 2 | 1.35E-10 | 4.50E-10 | 17.09 | Pantetheine,L-Valine |
| Phenylalanine metabolism | 10 | 1 | 4.32E-09 | 1.23E-08 | 18.35 | L-Phenylalanine |
| Phenylalanine, tyrosine and tryptophan biosynthesis | 4 | 1 | 4.32E-09 | 1.23E-08 | 18.35 | L-Phenylalanine |
| Arginine and proline metabolism | 38 | 2 | 6.39E-08 | 1.71E-07 | 17.02 | Creatine,1-Pyrroline-4-hydroxy-2-carboxylate |
| Glycine, serine and threonine metabolism | 33 | 1 | 7.05E-08 | 1.76E-07 | 17.02 | Creatine |
| Tryptophan metabolism | 41 | 3 | 9.42E-08 | 2.22E-07 | 15.51 | L-Tryptophan,Serotonin,L-Kynurenine |
| Folate biosynthesis | 27 | 1 | 3.14E-07 | 6.97E-07 | 16.17 | Sepiapterin |
| Sphingolipid metabolism | 21 | 4 | 3.36E-06 | 7.08E-06 | 14.03 | Sphingosine 1-phosphate,Sphinganine 1-phosphate,Sphingosine,3-Dehydrosphinganine |
| Thiamine metabolism | 7 | 1 | 6.60E-06 | 1.32E-05 | 14.04 | Thiamine |
| Lysine degradation | 25 | 1 | 1.96E-05 | 3.73E-05 | 13.13 | N6,N6,N6-Trimethyl-L-lysine |
| Galactose metabolism | 27 | 4 | 2.01E-04 | 3.10E-04 | 10.89 | Melibiose,D-Glucose,Galactitol,D-Mannose |
| Fructose and mannose metabolism | 20 | 1 | 2.02E-04 | 3.10E-04 | 10.89 | D-Mannose |
| Starch and sucrose metabolism | 18 | 1 | 2.02E-04 | 3.10E-04 | 10.89 | D-Glucose |
| Amino sugar and nucleotide sugar metabolism | 37 | 1 | 2.02E-04 | 3.10E-04 | 10.89 | D-Mannose |
| Neomycin, kanamycin and gentamicin biosynthesis | 2 | 1 | 2.02E-04 | 3.10E-04 | 10.89 | D-Glucose |
| Cysteine and methionine metabolism | 33 | 1 | 3.06E-04 | 4.53E-04 | 10.45 | L-Methionine |
| Steroid biosynthesis | 42 | 1 | 1.50E-03 | 2.14E-03 | 8.60 | Calcitriol |
| Porphyrin and chlorophyll metabolism | 30 | 2 | 7.34E-03 | 1.01E-02 | 6.19 | Biliverdin,D-Urobilinogen |
| Nicotinate and nicotinamide metabolism | 15 | 1 | 1.24E-01 | 1.65E-01 | 2.40 | N1-Methyl-2-pyridone-5-carboxamide |
| Biotin metabolism | 10 | 1 | 2.34E-01 | 3.02E-01 | 1.47 | Biotin |
| Tyrosine metabolism | 42 | 2 | 2.61E-01 | 3.26E-01 | 1.31 | Dopaquinone,Thyroxine |
| One carbon pool by folate | 9 | 1 | 3.30E-01 | 4.00E-01 | 0.99 | 10-Formyltetrahydrofolate |
| Caffeine metabolism | 10 | 3 | 6.50E-01 | 7.65E-01 | 0.22 | 1-Methylxanthine,Theobromine,Caffeine |
| Steroid hormone biosynthesis | 85 | 4 | 7.13E-01 | 8.15E-01 | 0.36 | Corticosterone,Cortisol,Androstenedione,18-Hydroxycorticosterone |
| Nitrogen metabolism | 6 | 2 | 8.63E-01 | 9.09E-01 | 0.03 | Carbamoyl phosphate,L-Glutamine |
| D-Glutamine and D-glutamate metabolism | 6 | 1 | 8.63E-01 | 9.09E-01 | 0.03 | L-Glutamine |
| Glyoxylate and dicarboxylate metabolism | 32 | 1 | 8.63E-01 | 9.09E-01 | 0.03 | L-Glutamine |
| Arginine biosynthesis | 14 | 3 | 9.25E-01 | 9.31E-01 | 0.08 | Carbamoyl phosphate,L-Glutamine,Urea |
| Pyrimidine metabolism | 39 | 5 | 9.31E-01 | 9.31E-01 | 0.03 | L-Glutamine,Carbamoyl phosphate,Uridine,Cytidine,Thymine |

Notes: *P*<0.05, significance; *P*<0.01, remarkable significance; ZA: ZJP after treatment; ZC: ZJP control as healthy people

**Table S4 Characteristic of MSEA in ZB and ZC**

| **Name** | **Total Compounds** | **Hits** | ***P*-value** | **FDR** | **Fold Enrichment** | **Hit Compounds** |
| --- | --- | --- | --- | --- | --- | --- |
| Purine metabolism | 65 | 7 | 4.23E-16 | 1.69E-14 | 21.69 | Xanthine,L-Glutamine,Adenosine,Hypoxanthine,Inosine,Adenine,Urea |
| Drug metabolism - other enzymes | 39 | 1 | 1.66E-14 | 3.32E-13 | 21.49 | Isonicotinic acid |
| Butanoate metabolism | 15 | 1 | 3.14E-14 | 4.19E-13 | 21.40 | 2-Hydroxyglutarate |
| Primary bile acid biosynthesis | 46 | 2 | 2.57E-13 | 2.37E-12 | 20.43 | Taurine,Cholic acid |
| Taurine and hypotaurine metabolism | 8 | 1 | 2.96E-13 | 2.37E-12 | 21.04 | Taurine |
| Aminoacyl-tRNA biosynthesis | 48 | 9 | 2.44E-12 | 1.63E-11 | 17.60 | L-Phenylalanine,L-Glutamine,L-Methionine,L-Valine,L-Alanine,L-Isoleucine,L-Leucine,L-Tryptophan,10-Formyltetrahydrofolate |
| Arachidonic acid metabolism | 36 | 3 | 2.12E-11 | 1.21E-10 | 15.34 | Leukotriene B4,Prostaglandin D2,5,6-DHET |
| Selenocompound metabolism | 20 | 1 | 2.40E-10 | 1.20E-09 | 19.41 | L-Alanine |
| Phenylalanine metabolism | 10 | 1 | 1.45E-09 | 5.81E-09 | 18.78 | L-Phenylalanine |
| Phenylalanine, tyrosine and tryptophan biosynthesis | 4 | 1 | 1.45E-09 | 5.81E-09 | 18.78 | L-Phenylalanine |
| Alanine, aspartate and glutamate metabolism | 28 | 3 | 2.23E-08 | 8.12E-08 | 13.11 | L-Alanine,L-Glutamine,Carbamoyl phosphate |
| Tryptophan metabolism | 41 | 3 | 1.72E-07 | 5.29E-07 | 15.10 | L-Tryptophan,Serotonin,L-Kynurenine |
| Pantothenate and CoA biosynthesis | 19 | 2 | 1.77E-07 | 5.29E-07 | 13.03 | Pantetheine,L-Valine |
| Valine, leucine and isoleucine degradation | 40 | 3 | 1.98E-07 | 5.29E-07 | 16.31 | L-Valine,L-Isoleucine,L-Leucine |
| Valine, leucine and isoleucine biosynthesis | 8 | 3 | 1.98E-07 | 5.29E-07 | 16.31 | L-Leucine,L-Isoleucine,L-Valine |
| Lysine degradation | 25 | 1 | 4.47E-07 | 1.07E-06 | 15.95 | N6,N6,N6-Trimethyl-L-lysine |
| Arginine and proline metabolism | 38 | 2 | 4.53E-07 | 1.07E-06 | 15.89 | Creatine,1-Pyrroline-4-hydroxy-2-carboxylate |
| Glycine, serine and threonine metabolism | 33 | 1 | 5.09E-07 | 1.13E-06 | 15.87 | Creatine |
| Sphingolipid metabolism | 21 | 4 | 3.92E-06 | 8.25E-06 | 13.63 | Sphingosine 1-phosphate,Sphinganine 1-phosphate,Sphingosine,3-Dehydrosphinganine |
| Thiamine metabolism | 7 | 1 | 1.48E-05 | 2.95E-05 | 13.38 | Thiamine |
| Galactose metabolism | 27 | 4 | 2.63E-05 | 4.22E-05 | 12.87 | Melibiose,D-Glucose,Galactitol,D-Mannose |
| Fructose and mannose metabolism | 20 | 1 | 2.64E-05 | 4.22E-05 | 12.87 | D-Mannose |
| Starch and sucrose metabolism | 18 | 1 | 2.64E-05 | 4.22E-05 | 12.87 | D-Glucose |
| Amino sugar and nucleotide sugar metabolism | 37 | 1 | 2.64E-05 | 4.22E-05 | 12.87 | D-Mannose |
| Neomycin, kanamycin and gentamicin biosynthesis | 2 | 1 | 2.64E-05 | 4.22E-05 | 12.87 | D-Glucose |
| Folate biosynthesis | 27 | 1 | 2.09E-03 | 3.22E-03 | 8.18 | Sepiapterin |
| Arginine biosynthesis | 14 | 3 | 4.43E-03 | 6.56E-03 | 4.72 | Carbamoyl phosphate,L-Glutamine,Urea |
| Nitrogen metabolism | 6 | 2 | 6.91E-03 | 9.04E-03 | 6.61 | Carbamoyl phosphate,L-Glutamine |
| D-Glutamine and D-glutamate metabolism | 6 | 1 | 6.91E-03 | 9.04E-03 | 6.61 | L-Glutamine |
| Glyoxylate and dicarboxylate metabolism | 32 | 1 | 6.91E-03 | 9.04E-03 | 6.61 | L-Glutamine |
| Pyrimidine metabolism | 39 | 5 | 7.00E-03 | 9.04E-03 | 6.41 | L-Glutamine,Carbamoyl phosphate,Uridine,Cytidine,Thymine |
| Steroid biosynthesis | 42 | 1 | 1.31E-02 | 1.64E-02 | 5.72 | Calcitriol |
| Cysteine and methionine metabolism | 33 | 1 | 2.45E-02 | 2.97E-02 | 4.82 | L-Methionine |
| Porphyrin and chlorophyll metabolism | 30 | 2 | 3.89E-02 | 4.57E-02 | 3.28 | Biliverdin,D-Urobilinogen |
| Tyrosine metabolism | 42 | 2 | 1.45E-01 | 1.65E-01 | 2.09 | Dopaquinone,Thyroxine |
| Biotin metabolism | 10 | 1 | 2.17E-01 | 2.41E-01 | 1.57 | Biotin |
| One carbon pool by folate | 9 | 1 | 3.98E-01 | 4.30E-01 | 0.75 | 10-Formyltetrahydrofolate |
| Nicotinate and nicotinamide metabolism | 15 | 1 | 4.74E-01 | 4.99E-01 | 0.54 | N1-Methyl-2-pyridone-5-carboxamide |
| Steroid hormone biosynthesis | 85 | 4 | 5.00E-01 | 5.13E-01 | 0.72 | Corticosterone,Cortisol,Androstenedione,18-Hydroxycorticosterone |
| Caffeine metabolism | 10 | 3 | 7.38E-01 | 7.38E-01 | 0.13 | 1-Methylxanthine,Theobromine,Caffeine |

Notes: *P*<0.05, significance; *P*<0.01, remarkable significance; ZB: ZJP before treatment; ZC: ZJP control as healthy people
